# Alexithymia and Insecure Attachment among Male Intimate Partner Violence Aggressors in the Dominican Republic

**DOI:** 10.3390/healthcare9121626

**Published:** 2021-11-24

**Authors:** Luis Vergés-Báez, David Lozano-Paniagua, Mar Requena-Mullor, Jessica García-González, Rafael García-Álvarez, Raquel Alarcón-Rodríguez

**Affiliations:** 1 Department of Psychology, The Catholic University of Santo Domingo, Santo Domingo 10108, Dominican Republic; norbertoverges@gmail.com; 2 Department of Nursing, Physiotherapy and Medicine, University of Almería, 04120 Almería, Spain; mrm047@ual.es (M.R.-M.); jgg145@ual.es (J.G.-G.); ralarcon@ual.es (R.A.-R.); 3 Faculty of Health Sciences, Human Sexuality Institute, Autonomous University of Santo Domingo, Santo Domingo 10103, Dominican Republic; raf.garcia@hotmail.com

**Keywords:** insecure attachment, avoidance, alexithymia, intimate partner violence, social sciences

## Abstract

The complexity of intimate partner violence and the impossibility of understanding it from single factors have been studied from different biological, psychological, and socio-cultural factors. A cross-sectional study was conducted on 187 men involved in legal proceedings for problems of violence in their intimate partner relationships in the Dominican Republic in order to explore whether insecure attachment represents a risk factor for alexithymia in men with violent behaviors. The attachment style was determinate by the Casullo and Fernández-Liporace Attachment Styles Scale, and alexithymia was assayed using the Latin American Consensual Toronto Alexithymia Scale (LAC TAS-20), a modification of the Toronto Alexithymia Scale (TAS-20). Chi-square test and multiple binary logistic regression analysis were performed to explore the phenomena of alexithymia and attachment styles in the context of a confinement center for male intimate partner offenders in the Dominican Republic. The results showed that insecure attachment represents a risk factor for alexithymia, being highest for avoidant attachment in the population studied. The results also highlight the influence of other factors such as education and maternal–familial relationships as a factor risk for alexithymia.

## 1. Introduction

Intimate partner violence (IPV), the most common form of violence that women experience globally, is an act of violence committed by a male intimate partner or ex-partner that results in either physical, sexual, or psychological harm directed towards females [1]. Traditionally, the population of male aggressors has been studied based on sociodemographic factors such as age, sex, academic level, and marital status. However, results in different groups have been inconsistent, and their relationship with violence in partner aggressors seems to be mediated by other factors [2,3,4]. According to current theories, the violent behavior within the partner has a multi-causal origin and has multiple associated risk factors. This multifactorial makes the study of these risk factors a difficult task [5]. According to the ecological theory of Heise, the IPV should be viewed as a multifactorial interaction among personal, situational and sociocultural factors [6]. Although both sexes are at risk of homicide from IPV, men are more likely than women to act violently in order to induce fear, domination, and control [7].

Violence in intimate relationships is a serious social problem in the Dominican Republic, as it is the most reported crime and claims many lives every year. According to official sources, in 2019, 152 women were murdered in the country in the intimate partner environment and 19,178 complaints were received. This number constitutes a rate of 2.7 per 100,000 women, the third-highest in Latin America, only surpassed by Honduras (6.2) and Salvador (3.3) [8]. In addition, from 2005 to 2020, 1514 women were murdered by partners and ex-partners in the Dominican Republic. Despite this reality, few studies have explored the possible relationships between the emotional factors present in offenders and their association with demographic and psychosocial variables, including alexithymia and attachment. However, the only sources considered for public policies are quantitative data on specific processes, leaving aside the qualitative relational and emotional aspects that could be associated with aggressor violence.

Attachment and alexithymia are emotional factors that have been regularly linked to violent behaviors in the male population and that have a direct impact on partner relationships. Although both have been studied in a possible relationship to male aggressors, they have been always studied separately [9,10,11].

Attachment could be defined as a special bond that constitutes an adaptive phenomenon. It is an organized behavioral system whose function is to maintain the proximity of care, and which acts as a homeostatic control system. Briefly, attachment theory establishes that both the resilience capacity of children and their subsequent behavior and emotional development are directly related to the type of bond that children established with their parents in the early years. According to this, attachment can be classified into two different styles: secure and insecure attachment (which can be divided into anxious and avoidant insecure attachment). Secure attachment occurs when the child feels unconditional on the part of her parents and has the certainty that they will not fail them. Within insecure attachments, for anxious attachment, the child does not trust their caregivers and grows up with a feeling of uncertainty and insecurity, which generates anguish. On the other hand, avoidant attachment occurs when caregivers do not provide sufficient security for the child, causing the child to develop an emotional distance from them [12].

Attachment is responsible for maintaining the stability of the individual in their environment [13]. In the avoidant attachment style, people feel uncomfortable being close to each other, while people with anxious attachment find that others do not come as close as they would like. In this sense, people with anxious attachment often feel that their partners do not love them, or even that they do not want to be with them [14,15]. Regarding the possible relationship between the style of insecure attachment and violence, some that theories have proven to be useful for predicting this phenomenon in relationships. Some evidence confirms that men with insecure attachment show greater violence against women than those with secure attachment [16]. Attachment styles in couples are measured through two dimensions, namely: anxiety concerning being abandoned and avoidance of intimacy [17]. In a general way, people with avoidable attachment tend to disable the attachment system by generating emotional distance in relationships. On the other hand, abusers who score high in anxious attachment often feel devalued in relationships and trigger a high level of violence [9,10,11]. For this reason, abusers with this attachment style are more likely to use violence to maintain emotional distance, as well as a mechanism to maintain control and revenge in relationships. 

On the other hand, alexithymia is a common personality trait among individuals with different health problems, suggesting that alexithymia could be a risk factor for medical or psychiatric illness [18]. Alexithymia could be defined as a personality trait in which people have serious difficulties identifying, describing, expressing, or verbalizing feelings. They also have difficulties differentiating bodily sensations and feelings during certain emotional experiences, particularly negative emotions. In addition, these individuals often present a restricted imagination, as well as a tendency to hostility when facing stressful situations [9,19]. Studies generally support the idea of a strong association between alexithymia and violence perpetrated by the aggressors due to the poor dyadic adjustment they have [9,19]. These studies also point out that individuals who have a higher level of insecure attachment style have a lower capacity to adequately display their emotional states, increasing their susceptibility to present alexithymia [18]. Previous studies link alexithymia and impulsivity, and use their association to explain aggressive behaviors and to establish the importance of emotional regulation [20].

Several demographic and psychosocial factors appear to be associated with alexithymia, although these findings appear to be inconsistent. The academic level, a factor that could reduce the possibilities of developing the emotional skills that alexithymia lack, seems to be the most determining factor [20,21,22]. The relationship between alexithymia and the quality of relationships, especially with the mother, should also be highlighted [23].

Previous studies have found that insecure attachment and alexithymia are associated with different kinds of violence in populations with mental health problems, such as depression, anxiety, somatoform disorders, personality disorders, or dissociative disorders [24,25,26]. The present study aims to study whether attachment styles could be a risk factor for alexithymia in male perpetrators of IPV confined in the Center for Behavioral Intervention for Male Aggressors in the Dominican Republic. As violence is also usually mediated by several factors, especially the quality of the parental relationship [10,27,28,29], the influence of this relationship has been also studied. Likewise, it intends to establish the socio-demographic profile of abusers with alexithymia in comparison with the non-alexithymia abuser‘s group.

## 2. Materials and Methods

### 2.1. Sampling and Data Collection

A cross-sectional study was conducted on a cohort of 187 men (male IPV aggressors) who were undergoing a therapeutic process at the Behavioral Intervention Center for Men of the Attorney General’s Office of the Dominican Republic as part of a coercive program for violence against their partners. Data were collected between January to June of 2019, at the beginning of the therapeutic program of the Center for Behavioral Intervention for Male Aggressors (early skills phase). Individuals selected were men over 18 years of age who had been detained at the Center during the previous year and voluntarily agreed to participate in the study. Participants had to be able to read and write and to have the capacity to understand the questions. Participants were not previously evaluated with the same instruments used in this study. A therapist interviewed all participants individually. There were two screening tests (The Casullo and Fernández-Liporace Scale and LAC TAS-20) that lasted an average time of 25 min each. In addition, according to institutional protocols, some questions about socio-demographic parameters were asked to obtain key information. 

Individuals who volunteered to participate in the study signed an informed consent form after being informed about the objectives of the study and their right to drop from the study at any time. The study was approved by the Bioethics Commission of the Institute of Human Sexuality belonging to the School of Medicine of the Autonomous University of Santo Domingo in the Dominican Republic. All the procedures were performed following the ethical standards of the Helsinki Declaration.

### 2.2. Instrumentalization

The evaluation began with the application of an interview to collect information on the socio-demographic characteristics of every participant. The questionnaire applied was designed by the Center for Behavioral Intervention for Men of the Attorney General’s Office of the Dominican Republic in 2008. This questionnaire was used since to collect information on the main variables such as age, marital status, occupation, educational level, type of violence exerted, and quality of the relationship with the mother. 

The Casullo and Fernández-Liporace Scale [13] was used to evaluate the attachment style that participants had. It is an adaptation of the original scale developed by Bartholomew in 1994 [30]. According to Bartolomew (1994), Casullo and Fernández-Liporace (2005) proposed a scale that integrates pathological attachments (fearful-avoidant and dismissing avoidant attachments) into a single category called avoidant attachment, as both share avoidance as a central aspect [13]. The test consisted of a scale on attachment styles considering three indicators (secure, anxious, and avoidant) from each of the four attachment styles originally mentioned by the author (secure, anxious, dismissing avoidant, and fearful avoidant). The test was divided into two parts: the first one was about romantic relationships (9 items) and the other one was on non-romantic relationships (11 items). Participants had to respond by scoring each item based on a Likert scale, in which the response values were sorted from lowest to highest in terms of frequency ((1) hardly ever; (2) sometimes; (3) frequently; (4) almost always). Items were presented as statements, and individuals had to respond to each according to which one best suited them. Three partial scores were obtained for each scale corresponding to the sum of the assigned values for each indicator. Direct scores were converted using statistical standards to percentiles. For the different styles of attachment, percentiles below 30 were considered low attachment, while percentiles above 70 reflected high attachment. In contrast, attachments with percentiles between 30 and 70 were related to medium attachment [13]. This scale featured a Cronbach’s alpha of 0.71. As three subscales were used to measure attachment, Cronbach’s alpha for each dimension was 0.62 for avoidant attachment; 0.51 for anxious attachment, and 0.85 for secure attachment.

To evaluate alexithymia, the Latin American Consensual Toronto Alexithymia Scale (LAC TAS-20) was used, for which the Spanish version of the Toronto Alexithymia Scale (TAS-20) was previously validated [31]. The alexithymia questionnaire consisted of three factors or subscales corresponding to each of the following theoretical dimensions: difficulty in identifying feelings and distinguishing them from the physiological sensations of emotions (DIF factor), difficulty describing other people’s feelings (DDF factor), and externally oriented thinking (EOT factor). The first factor, the DIF factor, consisted of seven items that evaluated the confusion of the interviewee about the emotions felt or the confusion about bodily sensations (items 1, 3, 6, 7, 9, 13, and 14). The DDF factor comprised five items that evaluated the ease or difficulty for finding words that described the feelings or emotions experienced by the interviewees (items 2, 4, 11, 12, and 17). The EOT factor was composed of eight items that evaluated external-oriented thinking, for example, the greater or lesser inclination to introduce sentimental topics into everyday conversations or the greater or lesser importance attached to reflection on the emotions experienced in everyday life (items 5, 8, 10, 15, 16, 18, 19, and 20).

Every question on the LAC TAS-20 was a Likert scale statement that could range between 1 and 5 points: (1) strongly disagree; (2) moderately disagree; (3) neither agree nor disagree; (4) moderately agree; (5) strongly agree. The evaluation of the scale is done by assigning the scores obtained. From the sum of these scores, the total score, and the score of each of the three factors (DIF, DDF, and EOT) could be obtained. Thus, the total score ranged from a minimum of 20 to a maximum of 100 points. Initially, the TAS-20 scale divided individuals into three groups: those scoring 56 points or more (definite alexithymia), those scoring between 41 and 55 (indefinite alexithymia), and those scoring less than 40 points (no alexithymia). However, for this study, this variable was dichotomized as the presence of alexithymia (scores equal to or above 40) or absence of alexithymia (scores below 40 points), as undefined alexithymia was considered no alexithymia being present. The scale presented a Cronbach’s alpha of 0.78. 

### 2.3. Data Analysis

A descriptive analysis of the continuous variables was carried out using means and standard deviations, whereas absolute and relative frequency distributions were calculated for categorical variables. Categorical variables were compared using the Chi-square test. Multiple binary logistic regression analysis was used to assess the risk of having alexithymia adjusted for variables that were considered to influence the statistical model based on the bivariate analysis. Insecure attachment (in the two manifestations: anxious and avoidant) was taken as an independent variable. The indicators of a warm relationship with the mother were predominance of affection, stimulus-centered education, and unconditional acceptance of the children. On the other hand, alexithymia was the dependent variable and was measured based on its presence or absence in the men evaluated. The level of statistical significance was established for a value of *p* < 0.05. The IBM-SPSS statistical software package (SPSS 26.0 for Windows) was used for all the statistical analyses.

## 3. Results

A total of 187 men receiving treatment for gender-based violence at the center of behavioral intervention for male aggressors participated in the study. These men were divided into two groups according to whether they had a previous diagnosis of alexithymia (*n* = 90) or not (*n* = 97). The study group did not differ regarding possible confounding factors such as age, marital status, period with the partner, employment status, and the number of children they had with the victims (Table 1). However, an association between the education level and the presence or absence of alexithymia was observed (*p* = 0.001). All participants were of Dominican nationality and the majority had medium studies (alexithymia 53.9%, *n* = 48; no alexithymia: 46.9%, *n* = 45; *p* < 0.001). Regarding the kind of violence perpetrated by the aggressors, the most predominant was psychological violence (55%), while physical violence was 45.5% (data not shown).

Table 2 shows the comparation between IPV perpetrators with and without alexithymia. Significant differences were observed for the avoidant and anxious attachment levels and for the maternal relationship, but not for the paternal relationship. In this sense, data reflected higher values of alexithymia for high avoidant attachment (34.1%), high anxious attachment (38.6%), and for a respectful relationship with the mother (51.2%). In contrast, alexithymia was less frequent for low avoidant attachment (9.1%), low anxious attachment (8.0%) and distant relation with mother (15.9%).

Table 3 shows the results of the multiple binary logistic regression analysis of the possible factors that may be associated with the development of alexithymia. The models were adjusted for the following independent variables: age, educational level, anxious attachment, avoidant attachment, and type of maternal relationship. A higher risk of having alexithymia was observed for aggressors with a medium level of avoidant attachment (OR = 6.82), for those who presented a medium level of anxious attachment (OR = 1.26), and for those who had a respectful maternal relationship (OR = 1.87).

## 4. Discussion

In this study, insecure attachment was analyzed as a risk factor for alexithymia in male aggressors. The study of these factors is necessary for a better understanding of the mechanism by which certain emotional factors (including alexithymia and attachment style) could influence the violent behavior of men with this condition. This association could be useful in programs for the re-education and reintegration of IPV offenders, proposing personalized programs according to the offender’s profile.

### 4.1. Attachment, Alexithymia, and Violence

The main finding of this study is that insecure attachment, in any of its manifestations, avoidant or anxious, represents a risk of alexithymia in the population of aggressor men studied. These results complement the findings of other research in which unsafe attachment suggests a risk of alexithymia in a different population [24,32,33]. Because alexithymia is an indicator of mental state, these findings are also consistent with Cramer’s statements that the quality of people’s mental state depends on the quality of the attachment [34].

Previous studies have established a possible relationship between the type of violence and certain behaviors lacking emotional regulation [35,36,37]. On this basis, several researchers establish that violence could be understood as a behavior in which a lack of emotional regulation is relevant [38]. Therefore, both insecure attachment and alexithymia could be associated with emotional dysregulation, leading to violence. This approach has been corroborated by several studies, which also report that alexithymia is more common in people with insecure attachment [33]. However, the results obtained in the present study do not allow for drawing any eventual causal relationships between attachment and alexithymia with IPV perpetration.

The indirect effect of attachment and alexithymia on violence and its mediating role through impulsivity have also been previously considered. The inability to regulate emotions is directly related to alexithymia, and alexithymia is directly related to impulsivity and violence [39,40]. The result of this study seems to be consistent with the evidence that raises the concurrence between alexithymia and emotional dysregulation with the inference that these conditions would relate to the insecure-based individual attachment style [41,42]. Another study [17] found evidence that confirms that the dynamics of attachment style operate as a risk factor for alexithymia from the traumatic experiences of childhood, thus confirming insecure attachment as an explanatory factor of emotional deregulation leading to violence. However, this association could not be confirmed by this study. The basis of these findings on childhood trauma and its association with violence in intimate relationships assumes that traumatic experience affects the biological, social, cognitive, behavioral dimensions, the attachment system, and health achievements, many of which are associated with criminal behaviors. Some research has found that people with alexithymia are more likely to demonstrate an inability to identify emotions. This lack of ability may be present in male aggressors in order to avoid verbalizing certain dysregulated emotions such as anger and fear. In this context, not being able to identify emotions complicates the regulation process and increases the possibility of resorting to violence [19].

In this study, although the direct relationship between alexithymia and violence has not been explored, a higher risk of alexithymia has been found in violent men when some manifestations of insecure attachment (distant or anxious) are present. Evidence confirms that safe attachment improves parental bonds and these, in turn, increase the indicators of emotional intelligence. The ability to identify emotions is strongly associated with the attachment style [43].

Both avoidant insecure and anxious insecure attachment are associated with alexithymia. The results confirm the associations between attachment and alexithymia in the population studied, but not the relationship of any of them with violence. A possible explanation for these results lies in the complexity of violent behavior in partner relationships, which makes them irreducible in its explanation of linear emotional processes. Therefore, the less likely the possibility of an agreement in the face of conflict leads alexithymic husbands to have lower satisfaction, less expression of affection, low cohesion, and poorer dyadic adjustment [19]. According to this, the link between alexithymia and violence in intimate relationships could be expected to have these variables as associated factors and, therefore, an emotional dysregulation could be capable of increasing the possibilities of violence [42].

### 4.2. Alexithymia and Quality of Relationship with the Mother

The quality of the relationship with parents, especially with the mother, has already been recognized as a protective factor for alexithymia [44]. In this study, this association was confirmed for respectful mother relationships. This finding is in accordance with several findings on attachment that predict better levels of emotional regulation and therefore higher alexithymia scores for people with insecure attachment [10]. Relationship quality and safe attachment have been consistent in different investigations [43,45]. This evidence is supported because people with a secure attachment style report greater maternal and parental warmth. However, maternal overprotection is not associated with a secure attachment style, nor with traumatic events or with experience of absences or separation for a long time from the mother. This last condition is negatively related to secure attachment.

Certain studies suggest that the deterioration of relationships with progenitors in early years negatively influences the model of relations that will be established later [46]. Although the aim of the study was not to show this relationship, it could be observed that alexithymia, as an emotional process, is associated with another emotional variable such as attachment style, which is also linked to early processes, such as the mental model of work that affects the quality of subsequent relationships. This model highlights that alexithymia is a by-product of a poor relationship with parents [47]. 

Certain studies also report that securely attached individuals have lower levels of psychological distress and highlight the poor experience of relationships with the mother in alexithymia problems [48]. Accordingly, it is logical to assume that, as deduced from the results of this study, attachment problems are associated with alexithymia and the quality of the maternal–filial relationship. Evidence confirms that security and basic trust in the relationship with parents from an early age represent much of the foundation for future emotional stability [46,49]. In this research, an association between alexithymia and the quality of the relationship with the mother was found in such a way that it confirms the trend of the above findings.

### 4.3. Alexithymia and Demographic Factors

The present study did not find an association between any of the demographic factors studied and alexithymia, except for the educational factor. Several studies have found an association between alexithymia and demographic variables such as age, marital status, occupation, and educational level, but most of them are inconsistent [3,4]. However, a previous study linking alexithymia and educational level [50] established that a high level of education could be considered a protective factor for alexithymia, as people with a low level of education have more difficulties in recognizing, distinguishing, or appreciating emotions than others with a higher level of education. In addition, people with a higher level of education have more awareness skills, as well as better indicators of emotional intelligence, which puts them in a better position to neutralize factors such as problems identifying emotions, descriptions, and processing, as well as working with their feelings, which are protective factors against alexithymia [50]. Given that some studies confirm that individuals with alexithymia may be prone to violence, including intimate partner violence against women, it is conceivable that level of education could be a variable that may influence the effects of relationship violence in addition to alexithymia [51]. However, this assertion should be validated by further studies that include a control group.

### 4.4. Strengths and Limitations of the Study

The main strength of the study was the exploration of the relevance of insecure attachment as a risk factor for alexithymia in a population of male abusers. This research highlights the importance of assessing alexithymia and attachment style in IPV perpetrators. The results of the study will encourage the search for therapeutic methods for an approach focused on male aggressors according to every personal attachment style. These interventions could have an impact on alexithymia, reducing the deregulatory effects as well as promoting strategies for emotional self-regulation, triggering a better adjustment for escalating situations that often lead to explosions.

Furthermore, this study promotes the insertion of instruments to measure attachment styles and the presence of alexithymia within the evaluations used in the initial stages of the attention programs for male aggressors. These instruments could be used for assessment purposes and in planning the treatment and rehabilitation plan for program participants.

However, the study also has several limitations. The main limitation is the lack of a control group, as only IPV perpetrators were included in the study, which avoids establishing a causal relationship comparing the rates between IPV perpetrators and those who were not. Another limitation is that, as the study was a cross-sectional study, it did not allow for establishing a causal linear relationship, depending on whether the insecure attachment determined the origin of the alexithymia. For this purpose, a longitudinal study should have been considered. On the other hand, some of questions raised were self-report responses and, as it was a population in conflict with the law, some individuals would appeal to social desirability in their responses. In addition, the study was carried out in an official center that belongs to the Dominican Republic’s Justice System, with a very limited study population, so the results obtained could not be extrapolated to the whole male population who have the status of perpetrators. The impossibility of measuring certain variables associated with IPV (sexism, marginality, myths about offenders, myths about battering within the couple, internalized social norms about masculinity, types of batterers, etc.) could be considered as other limitation of the study. It could lead to misinterpretation of the results and be taken as empirical support for some wrong myths (the wrong idea that IPV perpetrators have a mental illness or disorder that causes their violent behavior against their partners) that minimize the importance of IPV and could lead to exonerating them from responsibility [52]. In addition, another limitation could be the low reliability of the avoidant and anxious attachment measures [13].

## 5. Conclusions

The present study provides evidence on the relationship between the two forms of manifestations of insecure attachment (avoidant and anxious) and the condition of alexithymia in the aggressor population. On this basis, both alexithymia and insecure attachment could be related to the mechanism that explains certain violent behavior in intimate relationships and that it could be related to emotional factors, although it is not completely clear. This relationship allows us to propose the evaluation of alexithymia and personal attachment style as a therapeutic complement for the assessment of the male perpetrator’s profile that will support proposal rehabilitation plans adjusted to each profile. This study also highlighted the possibility that the presence of insecure attachment may represent a risk factor for alexithymia in the aggressor population. Finally, the present study also confirms that both maternal–filial relationships and a medium level of education are indicative of alexithymia among the population of IPV perpetrators studied. In this sense, quality mother–child relationships and high levels of education show a protective effect against the development of alexithymia. However, further research is necessary to confirm the conclusions that can be drawn from this study. Future research should consider the potential effects of including a control group more carefully.

## Figures and Tables

**Table 1:** Comparison of socio-demographic data between the alexithymia and no alexithymia groups.

Characteristics		Alexithymia (*n* = 90)	No Alexithymia (*n* = 97)	*p*-Value
Age (in Years)		38.50 (12.49)	36.80 (8.66)	0.28 ^a^
Marital status	Single	43 (48.9%)	48 (50.0%)	0.21 ^b^
	Non married	31 (35.2%)	28 (29.2%)	
	Married	14 (15.9%)	17 (17.7%)	
	Divorced	0	3 (3.1%)	
Employed	Yes	84 (93.3%)	88 (90.7%)	0.51 ^b^
	No	6 (6.7%)	9 (9.3%)	
Years in relationship	Engaged	5 (6.3%)	5 (6.3%)	0.61 ^b^
	One	6 (7.6%)	12 (15.0%)	
	2–5	25 (31.6%)	22 (27.7%)	
	6–9	25 (31.6%)	21 (26.3%)	
	10 or more	18 (22.8%)	20 (25.0%)	
Number of children with the victim	One	22 (27.7%)	17 (20.0%)	0.13 ^b^
	Two	20 (25.0%)	25 (29.4%)	
	Three or more	17 (21.3%)	10 (11.8%)	
	None	21 (26.3%)	33 (38.8%)	
Educational level	Low	23 (25.8%)	8 (8.3%)	0.001 ^b^
	Medium	48 (53.9%)	45 (46.9%)	
	High	18 (20.2%)	43 (44.8%)	

*p*-value obtained using the ^a^ Mann–Whitney *U* test for continuous variables or ^b^ Chi-squared test for categorical variables.

**Table 2:** Comparison of insecure attachment data (avoidant and anxious) and maternal and paternal relationship for both groups of study (alexithymia and non-alexithymia groups).

Variables		Alexithymia (*n* = 88)	No Alexithymia (*n* = 94)	*p*-Value *
Avoidant Attachment	Low	8 (9.1%)	26 (27.7%)	
	Medium	50 (56.8%)	57 (60.6%)	0.001
	High	30 (34.1%)	11 (11.7%)	
Anxious Attachment	Low	7 (8.0%)	10 (10.6%)	
	Medium	47 (53.4%)	64 (68.1%)	0.03
	High	34 (38.6%)	20 (21.3%)	
Relationship with mother	Warm	27 (32.9%)	55 (57.9%)	
	Respectful	42 (51.2%)	30 (31.6%)	0.004
	Distant	13 (15.9%)	10 (10.5%)	
Relationship with father	Warm	17 (21.0%)	32 (33.7)	
	Respectful	44 (54.3%)	43 (45.3%)	0.17
	Distant	20 (24.7%)	20 (21.1%)	

* *p*-value obtained using the Chi-squared test.

**Table 3:** Multiple binary logistic regression analysis of the parameters selected between aggressors with alexithymia and no alexithymia.

Parameters	OR	95% CI	*p*-Value
Avoidant Attachment (Medium)	6.82	2.13–21.79	0.001
Anxious Attachment (Medium)	1.26	1.32–4.96	0.001
Maternal Relationship (Respectful)	1.87	1.14–4.96	0.001

The regression model was adjusted for age, educational levels (0: high; 1: medium; 2: low), anxious attachment (0: high; 1: medium; 2: low), avoidant attachment (0: high; 1: medium; 2: low), maternal relationship (0: warm; 1: respectful; 2: distant). Goodness-of-fit for the Nagelkerke R-Square was 0.73 and the p-value for the Hosmer–Lemeshow test was 0.17.

## Data Availability

The data presented in this study are available on request from the corresponding author.
